# Population-level heterogeneity complicates utilization of *Synechococcus elongatus* PCC 7942 surface display platforms

**DOI:** 10.17912/micropub.biology.001097

**Published:** 2024-04-02

**Authors:** Lisa Yun, Jonathan K Sakkos, Daniel C Ducat

**Affiliations:** 1 DOE-MSU Plant Research Laboratories, Michigan State University, East Lansing, Michigan, United States; 2 Department of Biochemistry and Molecular Biology, Michigan State University, East Lansing, Michigan, United States

## Abstract

Surface display technologies have been primarily developed for heterotrophic microbes, leaving photosynthetic counterparts like cyanobacteria with limited molecular tools. Here, we expanded upon surface display systems in *Synechococcus elongatus* PCC 7942 by modifying two outer-membrane proteins, SomA and Intimin, to display tags ( *e.g.* , SpyTag) to mediate physical interactions of living cyanobacteria with other biotic and abiotic targets. While re-engineered SomA constructs successfully translocated to the cell surface and could bind to compatible ligands, the efficacy of the best-performing designs was limited by a poorly-understood heterogeneity in the accessibility of the tags in living cells, resulting in low attachment penetrance.

**Figure 1.**
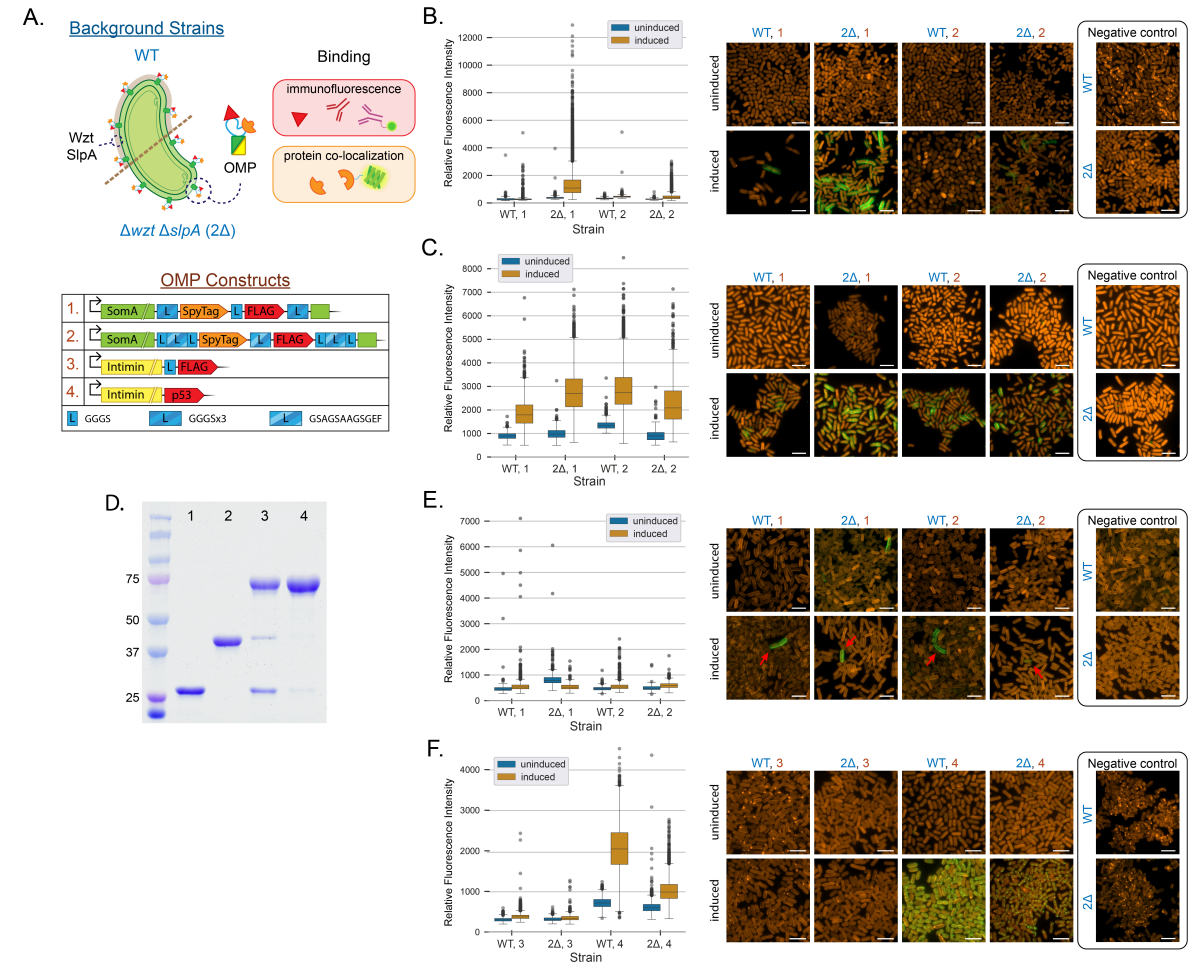
**A.** Schematic of background strains (WT and Δ *wzt* Δ *slpA* ) with engineered SomA or Intimin proteins with engineered binding domains expressed on the surface of *S. elongatus* . Surface domains were detected by immunofluorescence or protein co-localization, as indicated. The table below summarizes the linker and epitope/peptide modifications for each outer-membrane protein construct displayed in this figure. Immunofluorescence of fixed *S. elongatus* cells (constructs 1 and 2, as indicated) labeled with ( **B.** ) α-FLAG and ( **C.** ) α-SpyTag antibodies in cells bearing SomA-SpyTag-FLAG fusions. All constructs appeared to localize to the surface of *S. elongatus* when induced, albeit within a subset of the cell population that was dependent on strain background and the primary antibody used. **D.** SDS-PAGE of purified SpyTag-GFP (32.5 kDa; lane 1) and SpyCatcher-mNG (43.2 kDa; lane 2) after 10 and 120 minutes of *in vitro* binding (lanes 3 and 4, respectively). Nearly all of the SpyCatcher and SpyTag proteins are competent to form covalent bonds, as exhibited under the longer incubation periods. **E.** Protein co-localization of live *S. elongatus* cells (constructs 1 and 2) stripped with EDTA to remove surface-associated proteins and polysaccharides. Living cells were incubated with purified SpyCatcher-mNG and a small fraction of cells (generally <1%) exhibited localization patterns consistent with uniform binding of the reporter to SomA-SpyTag fusions (red arrows). **F.** Immunofluorescence of fixed *S. elongatus* cells (constructs 3 and 4) expressing Intimins fused to FLAG or p53 epitopes and labeled with α-FLAG or α-p53 antibodies, respectively. Cells exhibit limited expression (Intimin-FLAG) or mis-localization of expressed protein (Intimin-p53). All scale bars indicate 5 μm.

## Description

Surface display technologies offer a number of useful cellular properties that can be utilized for both academic research and biotechnological applications. However, these advancements have primarily been developed for only a few model heterotrophic microbes, leaving photosynthetic microbes, including cyanobacteria, with an underdeveloped molecular toolkit for surface display (Samuelson et al., 2002; Kondo and Ueda, 2004; Han et al., 2018) . The ability to engineer cellular surface properties and to customize the binding of biologically derived and inorganic materials to the outermost cell membrane leaflet holds the potential to introduce innovative strategies to overcome the challenges of large-scale cultivation of photosynthetic microbes. For example, biomass recovery and dewatering represent a major barrier to achieving economically competitive cultivation of microalgae and cyanobacteria, and surface display could offer new options for inexpensive harvesting (Vandamme et al., 2013; Mathimani and Mallick, 2018) .

In the current study, we set out to broaden the applicability of recently developed surface display systems for cyanobacteria to accelerate the potential of this important class of biotechnology chassis species (Santos-Merino et al., 2019, 2023) . Surface display of engineered epitope tags was reported in the model cyanobacterium, *Synechococcus elongatus* PCC 7942 (hereafter, *S. elongatus* ), to mediate binding to both biotic and abiotic targets (Chungjatupornchai and Fa-Aroonsawat, 2008; Chungjatupornchai and Fa-aroonsawat, 2009; Chungjatupornchai et al., 2011; Ferri et al., 2015; Fedeson and Ducat, 2017) . The primary strategy of these studies was to encode an epitope within an extracellular-facing loop of the endogenous outer-membrane porin protein, SomA. Initial designs showed some reactivity of engineered epitope protein tags ( *e.g.* , FLAG tag), but accessibility of the tags to appropriate antibodies was greatly improved by the genetic deletion of other factors likely occluding the cell surface, such as the O-antigen (OAg, *wzt* ) and the putative surface layer (S-layer, *slpA* ) proteins. Deletion strains (Δ *wzt* Δ *slpA* ) exhibit more accessible tags that interacted with antibodies targeting this specific epitope ( Fig. 1A ) and a similar improved penetrance of immunostaining was observed in cells treated with chelating agents known to strip away cell surface components (Fedeson and Ducat, 2017) .

To expand upon this toolkit, we evaluated multiple variants of the SomA outer-membrane porin and the heterologous adhesin, Intimin, in a range of genetic backgrounds of *S. elongatus* . Importantly, we sought to improve the utility of this surface display system by removing the requirement for specialized mediating antibodies for binding and to allow for direct adhesion. Towards this aim, we focused on two strategies: 1) inserting a SpyTag peptide for the covalent binding of its protein partner, SpyCatcher, into SomA at sites previously shown to allow for extracellular epitope tagging (Zakeri et al., 2012; Fedeson and Ducat, 2017) , and; 2) heterologous expression of Intimin proteins that form an extended surface spike-like projection for the specific purpose of attaching to other cells in *Escherichia coli,* and which have been modified with protein binding domains to mediate intercellular binding ( Figure 1A ; Glass & Riedel-Kruse, 2018; Robledo et al., 2022). In each approach we attempted multiple designs while varying the peptide linker length between domains, linker type ( *i.e.* , flexible vs. rigid), different binding motifs, and expressed these constructs in a wild-type (WT) or Δ *wzt* Δ *slpA* background of *S. elongatus* to control for any impacts of surface-occluding factors, though we will limit our discussion of the full range of variants tested due to meet formatting recommendations of this journal.

We evaluated expression, localization, and accessibility of the different surface display constructs using immunofluorescence and soluble reporter proteins fused to a compatible protein interaction domain. For surface display domains inserted in an extracellular facing loop of SomA ( Figure 1A, constructs 1 and 2), we first probed with α-FLAG antibodies in fixed cell populations. Immunostaining indicated a rise in the proportion of labeled cells following induced expression of the tagged SomA ( Figure 1B ). Furthermore, our results generally reinforced our prior observations that the engineered epitope tag was more accessible on the surface of the Δ *wzt* Δ *slpA* mutant background, relative to paired controls expressing the same construct in a WT background ( Figure 1BC ). However, significant intercellular variation of immunolabeling was observed and a proportion of cells in the induced cultures stained poorly or did not exhibit fluorescence at higher levels than the uninduced controls. Generally, constructs with longer linker sequences ( *e.g.,* GSAGSAAGSGEF) between introduced domains appeared to stain poorly with α-FLAG antibodies relative to constructs with shorter, flexible linker sequences ( Figure 1B ). Hypothetically, longer linker sequences may increase the distance between the epitope and the cell surface, but an extended linker may instead impose difficulty in translocating to the outer-membrane. In our best-performing SomA designs ( *i.e.* , GGGSx3 linker sequences in a Δ *wzt* Δ *slpA* background), the majority of fixed cells exhibited some staining localized to the cell periphery, although considerable variation in staining intensity was observed ( Figure 1B ). Due to the heterogenous staining, concerns regarding the integrity of the introduced SpyTag-FLAG epitope were raised, and we therefore conducted complementary immunostaining against SpyTag to validate if this epitope was localized to the cell surface. An extended incubation with α-SpyTag showed staining patterns similar to that of α-FLAG but with higher penetrance and average relative fluorescence intensity ( Figure 1C ). The majority of cells exhibited clear (albeit sometimes weak) staining with the α-SpyTag, with the exception of construct 2 in the WT background ( Figure 1C ). An overnight incubation with α-FLAG yielded a small handful of cells with puncta-like fluorescence on the cell periphery, whereas a 36-hour incubation with α-SpyTag reveals a spotty but more comprehensive labeling of the outer membrane.

To evaluate if the neighboring SpyTag domains were functionally active and accessible in the SomA fusions, we incubated cells in the presence of soluble SpyCatcher-mNeonGreen (mNG) reporter proteins ( Figure 1D ). SpyCatcher-mNG was purified and the majority of the protein formed covalent bonds with purified compatible SpyTag-GFP protein in a 10-minute *in vitro* incubation; the reaction was driven to near-completion in 2 hours ( Figure 1D ). When live cells bearing modified SomA-SpyTag constructs had their surfaces stripped with EDTA and incubated with the soluble SpyCatcher-mNG for 3 hours, a limited number of cells displayed a peripheral fluorescence signal consistent with successful binding between the SpyTag and SpyCatcher pair ( Figure 1E ); this staining pattern was never observed in negative control experiments using *S. elongatus* lacking an integrated SomA-SpyTag gene ( Figure 1E ). However, the vast majority of cells did not exhibit any SpyCatcher-mNG staining. Furthermore, there was no consistent trend in the proportion of cells exhibiting SpyCatcher-mNG attachment when considering induction of gene expression, various linker combinations, or the parental background in which SomA-SpyTag variants were expressed ( Figure 1E ). Nonetheless, the small fraction (<<1%) of labeled cells often displayed a clear localization pattern that could not be dismissed as a staining artifact ( Figure 1E ; red arrowheads).

Intercellular variation in the level of expression, localization, and accessibility of surface displayed domains was difficult to ascribe to any specific cellular feature or pattern. Relative to previously reported results (Fedeson and Ducat, 2017) , the SpyTag epitope was accessible at a comparable penetrance to when probed with a prolonged α-SpyTag antibody incubation. By contrast, the FLAG epitope showed lower accessibility as assessed by overnight with α-FLAG antibodies. Overall, there appeared to be poor correlation between cell lines with higher penetrance of modified SomA (as observed by fixed cell immunofluorescence, Figure 1B ) and the proportion of living cells that reacted strongly with soluble SpyCatcher ( Figure 1E ). The source of the intra-population variation in staining cells could not be explained as a merodiploid genetic insertion and was not resolved in separate control experiments using other engineered SomA variants, increased incubation times, or chemical protocols for ‘stripping’ the outer surface components with chelating agents (Sumper et al., 1990; Messner and Sleytr, 1992; Fedeson and Ducat, 2017) . Furthermore, SpyCatcher-reactive cells of the population exhibited chlorophyll *a* fluorescence ( Figure 1E ), and we were unable to find an association between cells exhibiting SpyCatcher-mNG staining and uptake of vital dyes; therefore, we cannot directly attribute the spurious staining with a subpopulation of dead cells. The nature of the variability remains largely unknown, although the most consistent explanation of our results is that other components (as yet, unknown) of the cell surface or extracellular matrix might contribute to epitope inaccessibility and the “all-or-nothing” staining patterns.

To evaluate if a separate surface display system could resolve the inconsistency, we expressed Intimin proteins that have been previously characterized to display a protein binding domain on an extended projection from the cell surface ( Figure 1A ; constructs 3 and 4). When expressed, Intimin-fusion proteins appeared to express poorly and/or primarily localize to the cytosol, as assessed by inclusion body-like immunofluorescence staining of fixed cell populations ( Figure 1F ). Consistent with these results, we did not observe any reactivity of living cells with α-FLAG or α-p53 antibodies above the level of paired negative controls. Our results appear to suggest that Intimins are not properly localized to the outer-membrane when expressed heterologously in *S. elongatus* .

In conclusion, while surface display technologies offer considerable biotechnological promise, our study highlights the complexities and challenges associated with achieving surface display of functional and accessible protein domains for direct adhesion in *S. elongatus* . There have been a limited number of successful reports of functional cyanobacterial surface display, such as the fusion of affibodies onto endogenous pili or S-layer proteins in *Synechocystis* sp. PCC 6803 and antibody-mediated adhesion in *S. elongatus* PCC 7942 (Cengic et al., 2018; Fedeson and Ducat, 2017) . Strikingly, while some living cyanobacteria expressing SomA-SpyTag fusions reacted strongly with soluble SpyCatcher and displayed localization patterns that would be expected from a functional surface display system, the vast majority of cells in the clonal population were non-reactive even under prolonged incubation and/or chemical treatment to strip surface-associated proteins and polysaccharides ( Figure 1E ). Despite genetic and chemical attempts to clear occlusions from the cell surface, expressing various linker types to extend further from the outer-membrane, and numerous variations to the binding procedures, we consistently observed low penetrance of binding to epitopes and SpyCatcher. One possible explanation is that the surface loops of SomA remain occluded by other endogenous cellular factors that project from the surface of *S. elongatus* , limiting accessibility even in surface-stripped cells. Attempts to remedy this possible obstacle by mounting epitopes on Intimins that project from the cell surface were unsuccessful, presumably due to a failure to correctly target these proteins to the outer-membrane ( Figure 1F ). It is possible that rare mutations in a minority of cells give rise to phenotypes with surface features amenable to display: selective recovery and sequencing of these rare cells could be revealing of the mechanism(s) responsible in this case.

Our results suggest that future surface display efforts may be more effective if they are focused on engineering targets that project further from the surface of the cyanobacterial outer membrane. For example, type IV pili could represent a promising avenue for further engineering, as they have been successfully employed for surface display purposes in *Synechocystis* sp. PCC 6803, and cell-encapsulating S-layer proteins can be utilized as a scaffold for high-density surface display rather than be removed as occlusions (Cengic et al., 2018; Charrier et al., 2019) . Ultimately, these results emphasize the need for further study into the outer-membrane of *S. elongatus* for better design and application of surface display technologies.

## Methods

**Cell culture and strain generation**

Cyanobacterial cultures were grown in baffled flasks in a Multitron Pro (Infors HT) incubator under constant illumination from fluorescent bulbs (150 μmol m ^−2^ s ^−1^ ; 15W Gro-Lux; Sylvania) in 32°C, 2% CO _2_ , and with 125 rpm shaking. Cultures were routinely grown in liquid BG-11 media supplemented with 1 g/L HEPES (H4034; Sigma-Aldrich) set to pH 8.3 with NaOH, with the appropriate antibiotic when needed (chloramphenicol, 25 μg/mL; kanamycin, 20 μg/mL; spectinomycin, 100 μg/mL). To improve consistency and maintain cultures in logarithmic growth, cultures for immunostaining or protein co-localization were back-diluted to an OD _750_ of ~0.3 each day for three days before inducing with 1 mM isopropyl-β-D-1-thiogalactopyranoside (IPTG; I2481C25; Goldbio). Cultures were induced for 24 hours prior to any subsequent cell staining or analysis.

The knockout background strain (Δ *wzt* Δ *slpA* ) was constructed by sequentially transforming with integration plasmids containing an antibiotic resistance selectable marker flanked by ~1 kilobase fragments upstream and downstream of the gene of interest on the 5’ and 3’ ends, respectively, as reported previously (Fedeson and Ducat, 2017) . Surface display strains were constructed by combining synthesized gene fragments of adhesins (Glass & Riedel-Kruse, 2018; Integrated DNA Technologies) and PCR-linearized NS3 integration vector with the *S. elongatus* SomA with Gibson Assembly (Gibson et al., 2009; Fedeson and Ducat, 2017) . Transformations were performed as outlined in Golden et al., 1987. Transformants were selected on BG-11 agar plates with appropriate antibiotics (chloramphenicol, 12.5 μg/mL; kanamycin, 16.7 μg/mL; and spectinomycin, 100 μg/mL), and verified by colony PCR and Sanger sequencing (RTSF Genomics Core, Michigan State University).

**Fluorescent protein expression, purification, and *in vitro* binding**

SpyCatcher-mNG and SpyTag-GFP plasmids were transformed into *E. coli* BL21(DE3) as is routine for protein purification, and transformants recovered on selective agar plates. Single colonies were cultured overnight at 37°C in 5 mL Luria-Bertani Broth (LB) with the appropriate antibiotic (ampicillin (50 μg/mL) for SpyCatcher-mNG, and kanamycin (50 μg/mL) for SpyTag-GFP). The following day, overnight cultures were back diluted 1:100 into fresh Terrific Broth (TB) with the appropriate antibiotic, and cultured at 37°C until a density of 0.4-0.6 OD _600_ was reached. At mid-log, cultures were chilled to approximately 23°C and induced with a final concentration of 400 μM IPTG for SpyCatcher-mNG and 500 μM anhydrous tetracycline (aTc) for SpyTag-GFP prior to culturing overnight at 23°C. Cell suspensions were chilled and transferred to a pre-weighed container to be pelleted, and the wet cell mass of the pellet was weighed before being resuspended with a minimal volume of lysis buffer and stored in -80°C until further processing. To prepare cells for lysing, frozen samples were thawed on ice and cold lysis buffer was added (final of 1:1 volume of lysis buffer to wet cell mass, w/w). During lysis, samples were kept on ice and a sonicator (Fisherbrand Model 120 Sonic Dismembrator; Fisher Scientific) equipped with a 1/8-inch probe microtip was used to disrupt cell integrity. Lysate was centrifuged 7,000 rpm for 25 minutes at 10°C on a Sorvall centrifuge equipped with the SS-34 fixed angle rotor (Sorvall) to pellet the cellular debris.

Expressed proteins (SpyTag-GFP and SpyCatcher-mNG) were recovered by affinity purification using encoded Hisx6 tags. To prepare the Ni-NTA agarose resin (Qiagen) for purification, 3 mL of the slurry was aliquoted into an empty gravity flow column (Poly-Prep Chromatography Columns; Bio-Rad) and the resin was washed 3 times with 3 mL lysis buffer. The cleared cell lysate was then loaded onto the column and the column was washed 4 times with 3 mL of the lysis buffer. Bound protein was eluted with the elution buffer. To remove the high concentration of imidazole from the elution buffer, the collected protein was transferred to an Amicon Ultra-15 Centrifugal Filter (Millipore) with the appropriate molecular weight cut off (20kDa for SpyCatcher-mNG and 10 kDa for SpyTag-GFP). The filters were centrifuged at 10°C to remove the bulk of the liquid before adding the protein storage buffer to dilute the remaining amount of imidazole before being centrifuged again; there were two total additions of protein storage buffer before the protein was collected, quantified, aliquoted, and flash frozen with liquid nitrogen.

To ascertain functionality, *in vitro* binding assays were performed. Approximately 230 pM of each SpyCatcher-mNG and SpyTag-GFP were diluted in phosphate citrate buffer (pH 7.5) before combining at room temperature. To stop the reactions for time points (10 and 120 minutes), samples were mixed 1:1 with 2X laemlli buffer (161-0737; Bio-Rad) and boiled at 95°C for 10 minutes. Samples were loaded onto 10% Mini-PROTEAN TGX Precast Protein Gels (Bio-Rad), and run with 75 V/10 mA for the first 10 minutes, and 120 V/30 mA for the remaining run time.

**Immunostaining and protein co-localization**

Cells were harvested (250 μL culture at ~1.5 OD _750_ ), pelleted, and washed in PBS. For immunofluorescence, cells were fixed, permeabilized, blocked, labeled with α-FLAG, α-SpyTag, or α-p53 antibodies, and labeled against the primary antibody with a fluorescent secondary antibody. To fix cells, washed pellets were resuspended with 200 μL pre-chilled methanol and incubated in -20˚C for 10 minutes. Methanol was removed, and cells were washed with 500 μL PBS three times. To permeabilize, fixed cells were resuspended with 300 μL of lysozyme solution, and incubated in 37˚C for 30 minutes. Lysozyme solution was removed, and cells were washed with 500 μL PBS three times. Permeabilized cells were then incubated with blocking buffer for 30 minutes at room temperature. Blocking buffer was removed, and cells were resuspended with 300 μL of a diluted primary antibody in blocking buffer (1:1000 dilution for α-FLAG (DYKDDDDK) (LT0420; Lifetein) and α-p53 (DO-1) Alexa Fluor 488 (sc-126 AF488; Santa Cruz Biotechnology), 1:5000 dilution for α-SpyTag (HCA406; Bio-Rad)). Samples with α-FLAG and α-p53 were incubated in 4˚C overnight, and α-SpyTag samples were incubated in 4˚C for ~36 hours. The primary antibody was removed, and cells were washed with 500 μL PBS three times before being resuspended in 500 μL blocking buffer and incubated at room temperature for 15 minutes. The blocking buffer was removed, and the samples were protected from light in the following steps until imaging. Cells were resuspended with 300 μL of a diluted secondary antibody in blocking buffer (α-mouse goat IgG DyLight™ 488 (35502; Thermo Fisher Scientific), or α-rabbit goat IgG DyLight™ 488 (35552; Thermo Fisher Scientific)), and incubated in 30˚C for 1 hour. After labeling, cells were pelleted, the secondary antibody removed, and cells were washed with 500 μL PBS three times. For protein co-localization, washed cells had the cell surface stripped by resuspending pellets with 200 μL 150 mM EDTA and incubated for 30 minutes at room temperature with end-to-end mixing. The EDTA solution was removed, and cells were washed with 500 μL phosphate citrate buffer three times.

To label living cells with the compatible soluble SpyCatcher-based reporter, cell pellets were collected as above, then resuspended with 300 μL phosphate citrate buffer with SpyCatcher-mNG (final molar ratio of ~1:20 SomA protein to SpyCatcher-mNG), and incubated for 3 hours with end-to-end mixing at room temperature while protected from the light. After labeling, the protein solution was removed, and cells were washed with 500 μL phosphate citrate buffer three times prior to imaging.

**Fluorescence microscopy and image analysis**

To image, labeled cells were resuspended with 15-30 μL phosphate citrate buffer or PBS, spotted onto agarose pads (1.5% w/v agarose in distilled water), and mounted onto glass coverslips. Fluorescence images were collected using an Axio Observer.D1 microscope (ZEISS), Axiocam 503 camera (ZEISS), 63x1.3 NA objective, X-Cite 120Q (Lumen Dynamics), and the Zen Blue (ZEISS) software. The following filter sets were used: filter set 43 (000000-1114-101; ZEISS) for chlorophyll autofluorescence, filter set 38 (000000-1031-346; ZEISS) for DyLight 488 fluorescence, and filter set 46 (000000-1031-346; ZEISS) for mNG fluorescence. Exposure times were held constant for fluorescent antibodies and SpyCatcher reporter proteins, respectively, so that outcomes could be compared across experiments.

Image analysis was performed with Python 3. Cell segmentation was conducted using custom Python scripts that used the Unet segmentation architecture (Ronneberger et al., 2015) for deep learning andimplementation by Pytorch (Paszke et al., 2019) . Mean fluorescence intensity plots were generated by segmenting the cells using the chlorophyll autofluorescence channel, rotating the cells such that the medial axis was horizontal, and rescaling the dimensions to 500 × 200 pixels to ensure consistent boundaries. The pixel intensity of the DyLight 488 and SpyCatcher-mNG was averaged from the segmented cells in the collection of images from each respective strain.

## Reagents

**Table:** 

**Plasmid name**	**Description**	**Source**
pDD493	Knockout plasmid to homologously recombine a Spc resistance selection marker in place of *wzt*	(Fedeson & Ducat, 2017)
pDD562	Knockout plasmid to homologously recombine a Kan resistance selection marker in place of *slpA*	(Fedeson & Ducat, 2017)
pDD963	NS3 integration vector with SomA-SpyTag-FLAG with three GGGS linkers before SpyTag and after FLAG tag, and a single linker in between the tags (construct 1); ChlR	This study
pDD996	NS3 integration vector with SomA-SpyTag-FLAG with a GSAGSAAGSGEF linker before SpyTag, after FLAG tag, and in between the tags (construct 2); ChlR	This study
pDD952	NS3 integration vector with Intimin-FLAG with a single GGGS linker in between the tags (construct 3); ChlR	This study
pDD954	NS3 integration vector with Intimin-p53 with no linker in between (construct 4); ChlR	This study
pDD1000	His-tagged SpyCatcher-mNG; AmpR	This study
pBbE2K-SpyTag-GFP-His6	His-tagged SpyTag-GFP; KanR	(Gonzalez-Esquer et al., 2021)

**Table:** 

***S. elongatus* strain name **	**Description**	**Source**
WT	Wild-type	
Δ *wzt* Δ *slpA*	Knockout of O-antigen (pDD493) and putative S-layer protein (pDD562)	(Fedeson & Ducat, 2017)
332	WT with construct 1 (pDD963)	This study
339	Δ *wzt* Δ *slpA* with construct 1 (pDD963)	This study
344	Δ *wzt* Δ *slpA* with construct 2 (pDD996)	This study
345	WT with construct 2 (pDD996)	This study
340	WT with construct 3 (pDD952)	This study
341	Δ *wzt* Δ *slpA* with construct 3 (pDD952)	This study
328	WT with construct 4 (pDD954)	This study
333	Δ *wzt* Δ *slpA* with construct 4 (pDD954)	This study

**Table:** 

*E. coli* strain name	Description	Source
SpyCatcher-mNG	BL21(DE3) with pDD1000	This study
SpyTag-GFP	BL21(DE3) with pBbE2K-SpyTag-GFP-His6	This study

**Table:** 

**Reagents used**	
BG-11	1X BG-11 media (C3061; Sigma-Aldrich) supplemented with 1 g/L HEPES (H4034; Sigma-Aldrich), pH 8.3
Phosphate buffered saline (PBS)	1 mM KH _2_ PO _4_ (3246-01; J.T. Baker), 1.5 mM NaCl (746398; Sigma-Aldrich), 5 mM Na _2_ HPO _4_ (3828-01; J.T. Baker), pH 7.4
Lysozyme solution	0.4 mg/mL lysozyme (0663; Amresco), 10 mM EDTA (0105; VWR International) in 10 mM Tris-HCl (15504-020; Invitrogen), pH 8
Blocking buffer	PBS, 5% (w/v) bovine serum albumin (126593; Merck Millipore)
Phosphate citrate buffer	40 mM Na _2_ HPO _4_ , 20 mM citric acid (251275; Sigma-Aldrich), pH 7.5
Lysis buffer	40 mM Na _2_ HPO _4_ , 20 mM citric acid, 150 mM NaCl, 5 mM imidazole (I2399; Sigma-Aldrich), 5% (v/v) glycerol (2136-03; J.T. Baker), 0.1% (v/v) TWEEN-20 (P9416; Sigma-Aldrich), 0.1 mg/mL lysozyme, 0.1 μg/mL DNase I recombinant (04536282001; Roche), 1X SigmaFAST protease inhibitor (S8820; Sigma-Aldrich)
Wash buffer	40 mM Na _2_ HPO _4_ , 20 mM citric acid, 250 mM NaCl, 10 mM imidazole
Elution buffer	40 mM Na _2_ HPO _4_ , 20 mM citric acid, 250 mM NaCl, 300 mM imidazole
Protein storage buffer	40 mM Na _2_ HPO _4_ , 20 mM citric acid, 250 mM NaCl, 10% (v/v) glycerol, 0.1% (w/v) sodium azide (S2002; Sigma-Aldrich)
